# Distinct respiratory microbiota associates with lung cancer clinicopathological characteristics

**DOI:** 10.3389/fonc.2023.847182

**Published:** 2023-02-02

**Authors:** Xi Zheng, Xingbing Lu, Yang Hu

**Affiliations:** ^1^ Lung Cancer Center, West China Hospital, Sichuan University, Chengdu, China; ^2^ Department of Laboratory Medicine, West China Hospital, Sichuan University, Chengdu, China; ^3^ Department of Thoracic surgery, West China Hospital, Sichuan University, Chengdu, China

**Keywords:** respiratory microbiota, lung cancer, pathology, antibiotic, smoking

## Abstract

**Introduction:**

Commensal microbiota dysbiosis is associated with the development of lung cancer. The current studies about composition of respiratory microbiota in lung cancer patients yielded inconsistent results. This study aimed to examine the association between airway microbiota and lung cancer clinicopathological characteristics.

**Methods:**

Surgically removed lesion tissues from 75 non-small cell lung cancer patients and 7 patients with benign pulmonary diseases were analyzed by 16S rRNA sequencing. Taxonomy, relative abundance, and diversity of respiratory microbiota were compared among lung cancer of different pathology and TNM stages. The effects of antibiotic and cigarette exposure on respiratory microbiota in lung cancer patients were also evaluated.

**Results:**

Bacterial relative abundance and alpha- and beta-diversity analysis of lung microbiota showed significant differences among lung cancer of different pathology and benign pulmonary diseases. At the genus level, the abundance differences of 13 taxa between lung squamous cell carcinoma and lung adenocarcinoma, 63 taxa between lung squamous cell carcinoma and benign pulmonary diseases, and 4 taxa between lung adenocarcinoma and benign pulmonary diseases reached statistical significance. In contrast, diversity differences were not as significant across lung cancer of different stages. No significant differences were observed in tissue taxonomic abundances and diversity at all taxonomic levels between lung cancer patients with and without antibiotic exposure 3 months prior to surgery. For lung adenocarcinoma, respiratory bacterial abundance and diversity at all taxonomic levels did not show significant differences between smokers and non-smokers.

**Conclusions:**

Our results confirm significantly differential respiratory microbiome taxa, abundance, and diversity in lung cancer of different pathology and some stages. Short-term antibiotic application might play a minor role in molding airway microbiota in lung cancer patients. Composition and diversity of respiratory microbiota in lung adenocarcinoma are not affected by cigarette exposure.

## Graphical Abstract

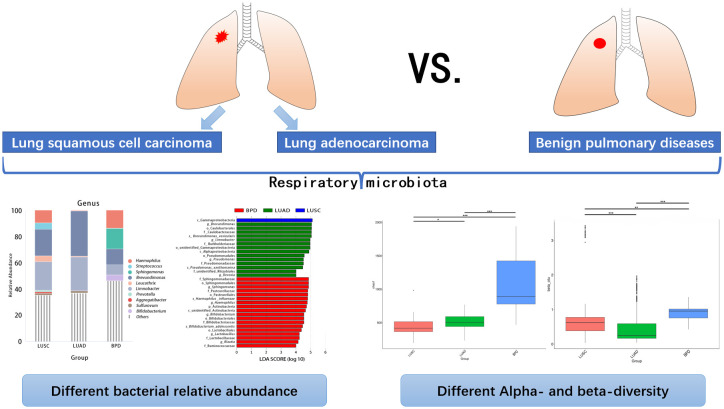

## 1 Introduction

Lung cancer remains the cardinal cause of cancer-related deaths worldwide (1). Main treatment options for lung cancer include surgery, radiotherapy, chemotherapy, driver gene-targeted therapy, and the newly emerged immunotherapy. Patients with early-stage lung cancer usually have satisfactory prognosis after treatments, but most patients have advanced diseases at their first diagnosis and missed the opportunity for curative treatments. The overall 5-year survival rate of lung cancer is still less than 20% (2).

Microbiota refers to all microorganisms that exist in human body sites such as skin, the digestive tract, urogenital tract, and airway. Approximately 10–100 trillion microorganisms live in the human body, and the number of their cells and genes are far more than that of human hosts themselves (3). Microbiota participate in various physiological processes including digestion, metabolism, immune system maturation, defense of pathogens invasion, and the differentiation and gene expression of mucosal epithelium (4). Dysbiosis in the current study refers broadly to disruption of microbial homeostasis with compositional or functional deviation from microbiota seen in healthy people without lung disease (5). As a part of tumor stroma, dysbiosis of commensal microbiota is associated with development of various malignant tumors and about 13% of cancer cases were driven by microbial pathogen. (6). The luminal microorganisms may affect cancer progression by changing the permeability of the mucosal barrier, activating inflammatory pathways, producing bacterial toxins that impair host genome stability, releasing cancer-promoting metabolites, and modulating local immune microenvironment (7).

As the widest mucosa-coated tissue in the human body, the respiratory tract is continuously exposed to the oropharynx, microorganisms in the air, and other damaging factors. The traditional theory based on bacterial culture techniques deems a healthy respiratory tract as sterile. However, high-throughput sequencing of the airway specimens indicates that the respiratory tract has a low biomass and diverse microbiota (8). Compared with gut microbiota, the airway microbiota has distinct microbial structures and microenvironment (8–10). The disturbance of airway microbiota is involved in the pathogenesis of various respiratory diseases including pneumonia, asthma, cystic fibrosis, chronic obstructive pulmonary diseases, and bronchiectasis (11). Recent studies also indicated that the initiation and progression of lung cancer were related to respiratory microbiota (7, 12, 13). Although dysbiosis of gut microbiota is observed in lung cancer patients, the local bacterial burden of the respiratory tract, rather than the gut, is correlated with the progression of lung tumors (13, 14).

Our group previously demonstrated that, compared with benign pulmonary diseases and healthy controls, dysbiosis of respiratory microbiota was observed in lung cancer patients. Certain bacterial taxa might be used as potential biomarkers for lung cancer (10). Here, we extend our work to lung cancer patients with different staging and pathology to examine the association of airway microbiota with clinical characteristics of lung cancer. We hypothesized the presence of distinct airway microbiota among lung cancer patients with different TNM staging and pathological types. The current study also tried to provide preliminary lines of evidence about the potential roles of respiratory microbiota in the pathobiology of lung cancer.

## 2 Materials and methods

### 2.1 Study design and participants

The current observational cohort study recruited inpatients with a high-resolution CT-confirmed diagnosis of pulmonary mass or nodules ready for surgery at our institution. From 2 January 2020 to 28 February 2020, 0.5 cm^3^ (average size) surgically resected lesions from the lower part of the lungs of 76 lung cancer patients (adenocarcinoma and squamous cell carcinoma) and 10 patients with benign pulmonary diseases without infection symptoms were consecutively collected for 16S rRNA biomarker sequencing in this study. Enrolled patients gave written informed consent and the study protocol was approved by the Institutional Review Board committee of our hospital. Among the included cancer patients, none received neoadjuvant therapy. For adenocarcinoma, microinvasive and invasive adenocarcinoma were included, and preinvasive lesions (atypical adenomatous hyperplasia, carcinoma *in situ*) or infrequent histological types (e.g., mucinous adenocarcinoma, colloid adenocarcinoma, fetal adenocarcinoma, and intestinal-type adenocarcinoma) were excluded. After quality control, one tumor tissue and three benign lesion samples were excluded from further analysis due to failed 16S PCR amplification. In the final analysis, 30 squamous cell carcinoma samples were included in the LUSC group, 45 adenocarcinoma samples were included in the LUAD group, and 7 benign pulmonary lesions were included in the BPD group. The BPD cases were composed of three inflammatory pseudotumor and four tuberculoma. Participants with lung cancer were older than those with benign pulmonary diseases. The percentages of male smoking individuals were higher in the squamous cell carcinoma group. Degree of differentiation and TNM stage between the two different lung cancer histology were also different. Other clinical characteristics were similar (**Table 1**).

**Table 1:** Demographic and clinical characteristics of study participants.

Characteristics		Squamous cell carcinoma	Adenocarcinoma	Benign pulmonary diseases	*p*-value
Age (years), mean (SD)		61.70 (8.90)	58.98 (9.92)	51.71 (5.22)	0.039
Male sex		30 (100.00)	26 (57.78)	3 (42.86)	<0.001
Smoking status (yes), n (%)		29 (96.67)	14 (31.11)	3 (42.86)	<0.001
Diabetes (yes), n (%)		6 (20.00)	6 (13.33)	1 (14.29)	0.741
Hypertension (yes), n (%)		7 (23.33)	10 (22.22)	1 (14.29)	0.861
COPD (yes), n (%)		5 (16.67)	4 (8.89)	1 (14.29)	0.595
Recent antibiotic consumption (yes), n (%)^a^		10 (33.33)	23 (51.11)	1 (14.29)	0.129
Differentiation, n (%)^b,c^	Well Median Poor	2 (6.90) 7 (24.14) 20 (68.96)	6 (16.22) 24 (64.86) 7 (18.92)	NA NA NA	<0.001
TNM stage n (%)^d,e,f^	I IIIII	8 (26.67) 9 (30.00)13 (43.33)	32 (72.73) 4 (9.09)8 (18.18)	NA NANA	<0.001

a Antibiotic exposure within 3 months prior to the lung lesion resection.

b One case of squamous cell carcinoma in situ was excluded.

c One case of adenocarcinoma in situ and seven cases of microinvasive adenocarcinoma were excluded.

d The eighth edition of the TNM classification for lung cancer (15).

e One case of stage Iva adenocarcinoma was excluded in the comparison of TNM stages.

f Nine patients in stage I, three in stage II, and four in stage III administered antibiotics within 3 months prior to the lung lesion resection.

### 2.2 Sample preparation and 16S rRNA gene sequencing

The resected pulmonary mass or nodule tissues were obtained and stored in sterile vials in liquid nitrogen under aseptic conditions. Diagnoses were verified by histopathology according to WHO histological guidelines (16). Empty vials were set as blank controls to reduce potential contaminations. The CTAB/SDS method was used to extract the total genome DNA in samples, and the DNA concentration and purity were assessed by 1% agarose electrophoresis (17). Then, DNA was diluted to 1 ng/μl using sterile water according to the concentration.

The bacterial 16S rRNA V4 region was amplified using the universal primer that targets this region in most bacteria (forward primer: 5′-GTGCCAGCMGCCGCGGTAA-3′ and reverse primer: 5′-GGACTACHVGGGTWTCTAAT-3′) with the barcode (18). All polymerase chain reactions were carried out with 15 µl of Phusion^®^ High-Fidelity PCR Master Mix (New England Biolabs), 0.2 µM of forward and reverse primers, and 10 ng of template DNA. Thermal cycling consisted of initial denaturation (98°C for 1 min), 30 cycles of denaturation (98°C for 10 s), annealing (50°C for 30 s), elongation (72°C for 30 s), and finally 72°C for 5 min. The same volume of 1× loading buffer was mixed with PCR products and electrophoresed on 2% agarose gel for detection. PCR amplicons were mixed in equidensity ratios. Then, the Qiagen Gel Extraction Kit (Qiagen, Germany) was used to purify the PCR amplicon mixtures.

A TruSeq^®^ DNA PCR-Free Sample Preparation Kit (Illumina, USA) with index codes was used to generate sequencing libraries following the manufacturer’s instructions. The Qubit@ 2.0 Fluorometer (Thermo Scientific) and Agilent Bioanalyzer 2100 system were used to evaluate the library quality. Lastly, the library was sequenced and 250-bp paired-end reads were generated (Illumina NovaSeq 6000 platform).

2.3. Statistical and bioinformatic analysis

Statistical analysis was done using R (version 2.15.3) software and SPSS (Version 17.0) as previously described (19). Continuous data were compared using median and interquartile range, while categorical variables were analyzed by the frequency and percentage in each category. Differences between groups were evaluated using ANOVA, the Kruskal–Wallis test, or the Wilcoxon matched pairs signed rank test.

The study shared the same statistical analysis in R and pipelines in QIIME with the previous study conducted by Rea Bingula et al. (20). Briefly, paired-end reads were assigned to samples based on their unique barcode and truncated by cutting off the barcode and primer sequence. FLASH (V1.2.7, http://ccb.jhu.edu/software/FLASH/) software was used to merge paired-end reads. The raw tags were filtered under default conditions to obtain the high-quality clean tags according to the QIIME (V1.9.1, http://qiime.org/scripts/split_libraries_fastq.html) quality control process. The tags were then compared with the reference database (Silva database, https://www.arb-silva.de/) using the UCHIME algorithm (UCHIME Algorithm, http://www.drive5.com/usearch/manual/uchime_algo.html) to detect and remove chimera sequences.

Uparse software (Uparse v7.0.1001, http://drive5.com/uparse/) was used to analyze sequences. Sequences with no less than 97% similarity were assigned to the same OTUs. The representative sequence of each operational taxonomic unit (OTU) was filtered for further annotation. The taxonomic information of each representative sequence was annotated *via* the Silva Ref NR Database (http://www.arb-silva.de/) based on the Mothur algorithm. MUSCLE software (Version 3.8.31, http://www.drive5.com/muscle/) was used to align multiple sequences to study the phylogenetic relationship of different OTUs and the difference of the dominant species in different samples. The sequence number of the sample with the least sequences was set as a standard to normalize OTUs’ abundance information. Subsequent analysis of alpha-diversity and beta-diversity were all performed based on this output normalized data.

Complexity of species diversity for a sample was evaluated by alpha-diversity through three indices, namely, observed species, Chao1, and Shannon. All these indices were calculated with QIIME and displayed with R software. Differences of samples in species complexity were assessed by beta-diversity analysis, which was also calculated by QIIME software through the index, weighted UniFrac. Cluster analysis was done by principal component analysis (PCA) using the FactoMineR and ggplot2 packages in R software. Principal coordinate analysis (PCoA) was performed to get principal coordinates and visualize multidimensional data. PCoA analysis was displayed by WGCNA, stat, and ggplot2 packages in R software. Unweighted pair-group method with arithmetic means (UPGMA) clustering was calculated by QIIME software as a type of hierarchical clustering method to interpret the distance matrix using average linkage. The detailed software and the corresponding parameters used in the 16S rRNA amplicon analysis in our study are listed in **Table S1**.

## 3 Results

### 3.1 The taxonomic diversity of respiratory microbiota in lung cancer and benign pulmonary lesion tissues

**Figure 1:**
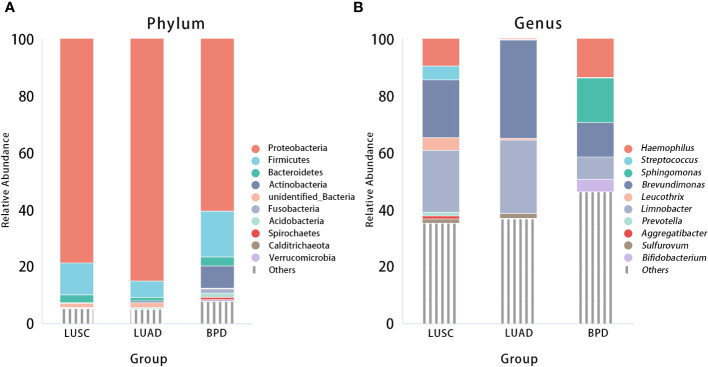
Relative abundance of respiratory microbiota composition at the phylum **(A)** and genus **(B)** levels by pathological statuses (LUSC *vs*. LUAD *vs*. BPD).

**Figure 2:**
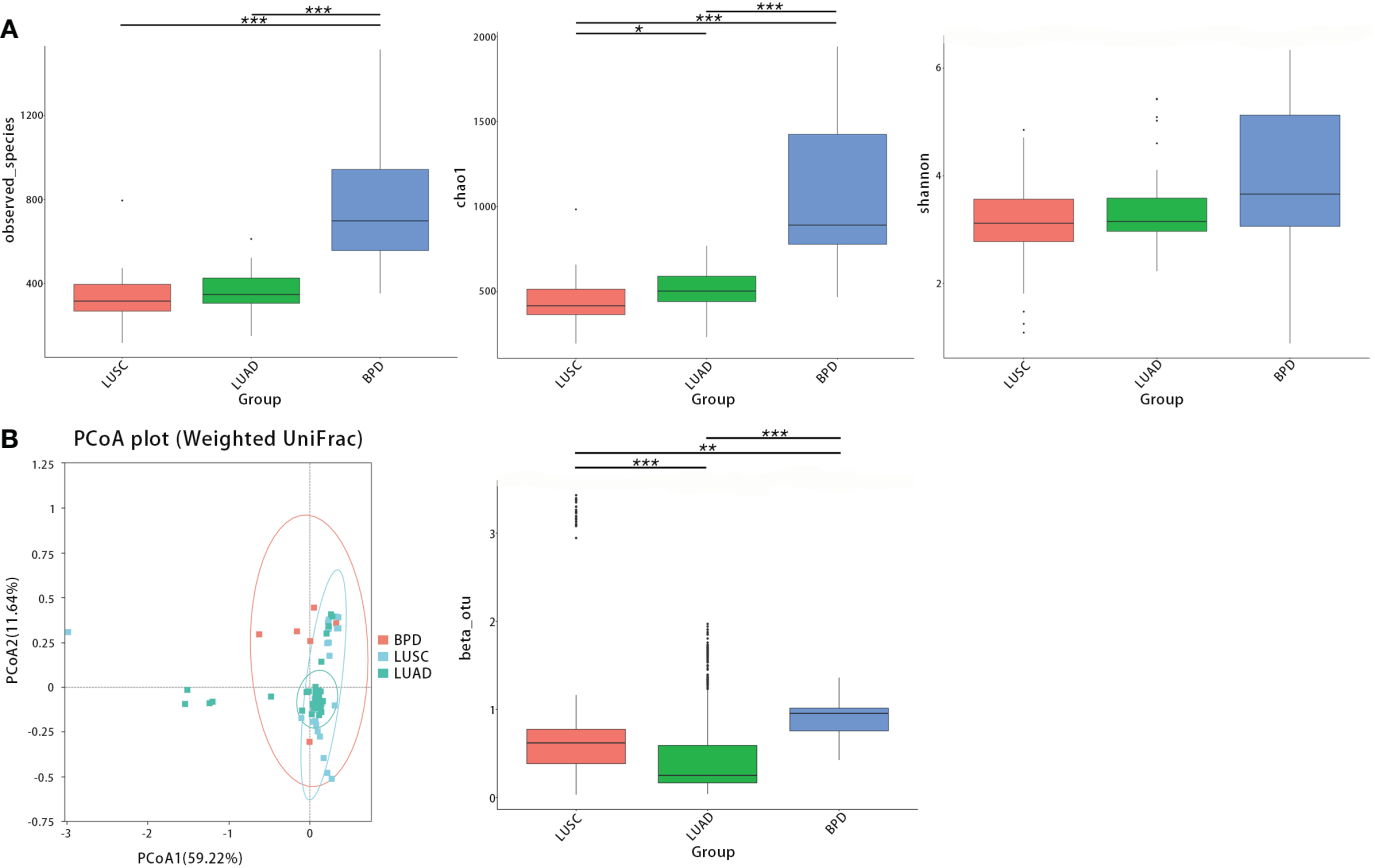
The taxonomic diversity of respiratory microbiota by group (LUSC *vs*. LUAD *vs*. BPD). **(A)** Significant differences in observed OTUs and Chao1 were observed between each group. No significant difference was observed between each group regarding Shannon’s index. **(B)** Principal coordinate analysis (genus level) using the weighted UniFrac distance displayed the dissimilarities of respiratory microbiota between each group. (*p < 0.05, **p < 0.01, and ***p < 0.001, similarly hereinafter).

After filtering and rarefying, a median of 63,381 high-quality reads were attained per tissue sample undergoing 16S rRNA sequencing (range = 35,036–69,704 tags). Based on 97% sequence similarity, the above reads were binned into 6,392 OTUs. According to taxon annotation results of all OTUs, the top four phyla in LUSC, LUAD, and BPD groups included Proteobacteria (78.60%, 84.91%, and 60.47%, respectively), Firmicutes (11.25%, 5.72%, and 16.09%, respectively), Bacteroidetes (2.71%, 1.28%, and 3.22%, respectively), and Actinobacteria (0.28%, 0.73%, and 7.70%, respectively) (**Figure 1A**). The predominant genera in the above three groups included *Haemophilus* (9.54%, 0.20%, and 13.59%, respectively), *Streptococcus* (4.82%, 0.17%, and 0.19%), *Sphingomonas* (0.03%, 0.10%, and 15.70%, respectively), and *Brevundimonas* (20.29%, 34.32%, and 12.04%, respectively) (**Figure 1B**). The top 30 taxa of relative abundance in each group are listed in **Table S2**. Then, the differences in taxonomic diversity of respiratory microbiota in lung cancer and benign pulmonary lesion tissues were analyzed. ANOSIM (analysis of similarities) is mainly used to analyze inter-group similarity of high-dimensional data and provide a basis for inter-group difference evaluation (21). ANOSIM suggested that significant differences existed in LUSC, LUAD, and BPD groups (**Table S3**). Alpha-diversity describes the richness and evenness of bacteria in samples. To evaluate differences in respiratory microbial alpha-diversity between lung cancer and benign pulmonary diseases, the diversity and richness indices were compared. A comparison of the observed OTUs and Chao1 presented a significant reduction in lung cancer groups (**Figure 2A**; **Table S3**). However, no statistical significance in Shannon’s diversity index was observed (**Table S3**). To compare the extent of dissimilarity in respiratory microbial composition between each group, beta-diversity was computed based on the weighted UniFrac distance. The PCoA plot was used for presenting the microbial composition of each group, and Wilcoxon analysis was performed to compare the dispersion among pathological statuses. The PCoA plot showed significant differences in clustering of each group (**Figure 2B**; **Table S3**).

**Figure 3:**
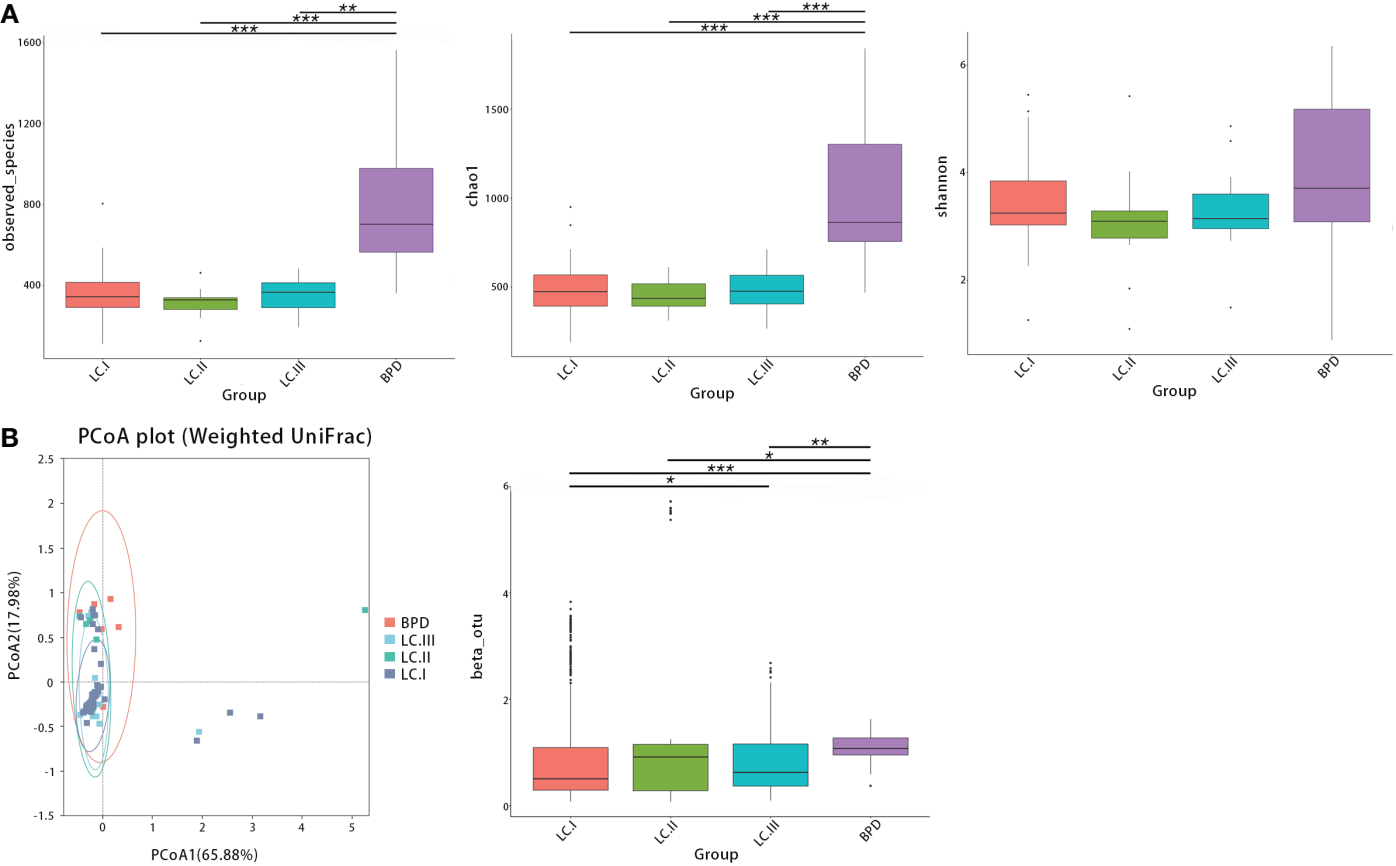
The alpha- and beta-diversity of respiratory microbiota in lung cancer patients by different TNM stages [stage I (LC.I) *vs*. stage II (LC.II) *vs*. stage III (LC.III)]. **(A)** No significant differences in observed OTUs, Shannon, and Chao1 were observed between different lung cancer TNM stages. Significant differences were observed between benign pulmonary diseases (BPD) and all stage I–III lung cancer cases regarding observed OTUs and Chao1 richness estimator. **(B)** Principal coordinate analysis (genus level) using the weighted UniFrac distance showed similar clusters of respiratory microbiota between lung cancer at different TNM staging and different clusters were found between benign pulmonary diseases and all lung cancer cases. * means 0.01 ≤ P < 0.05; ** means 0.001 ≤ P < 0.01; *** means P < 0.001.

Next, we further evaluated differences in respiratory microbial alpha- and beta-diversity between different pathological stages. LUSC and LUAD groups were lumped together in the comparisons by lung cancer stages. ANOSIM also suggested that differences between groups were significant (**Table S3**). The observed OTUs, Shannon, and Chao1, all indicated that there were no statistical significances between bacterial alpha diversity of different lung cancer TNM stages (**Figure 3A**). In contrast, compared with benign pulmonary diseases, the observed OTUs and Chao1 richness estimator were significantly lower in all stage I–III lung cancer lesions (**Figure 3A**; **Table S4**). For beta-diversity (weighted UniFrac distance), a similar trend was observed. Namely, different clusters were presented between benign pulmonary diseases and all lung cancer cases, and cluster differences between different lung cancer stages were not as significant (**Figure 3B**; **Table S4**).

### 3.2 Comparison of the respiratory microbial taxa in tissues of lung cancer and benign pulmonary diseases

**Figure 4:**
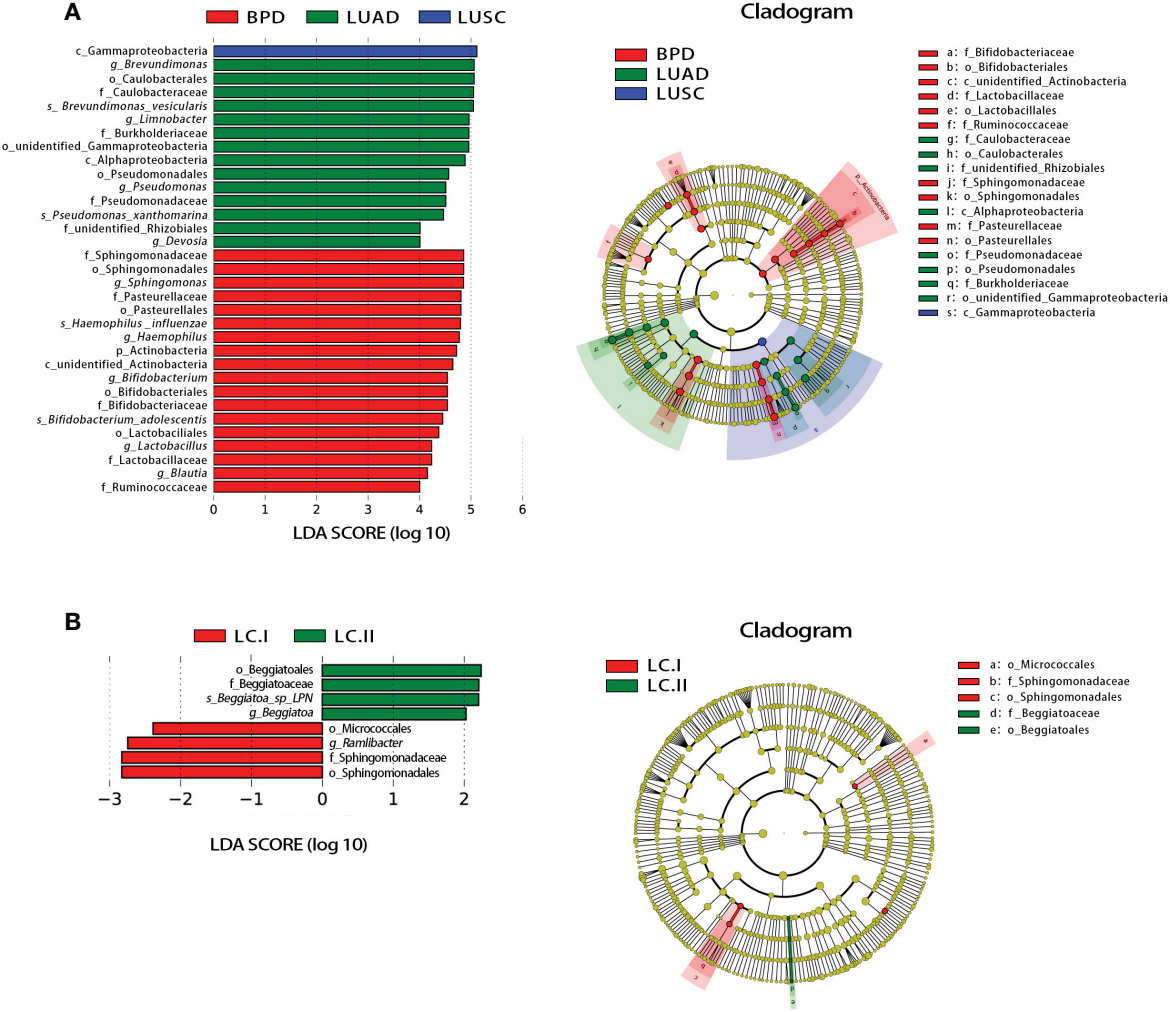
Linear discriminant analysis (LDA) effect size (LEfSe) analysis for respiratory microbiota abundance based on lung lesion pathology and lung cancer TNM staging. **(A)** The LDA scores (>4) and cladogram of the representative taxa associated with lung cancer and BPD. **(B)** The LDA scores (>2) and cladogram of the representative taxa associated with different lung cancer TNM stages.

Significant differences based on FDR (false discovery rate) correction in tissue taxonomic abundances at various taxonomic levels were observed between lung cancer and benign pulmonary diseases. At the phylum level, the abundance of Dadabacteria and Planctomycetes was different between lung adenocarcinoma and BPD (**Table S5**). No bacterial taxa showed significant abundance difference among different lung cancer stages at the phylum level (**Table S5**). At the genus level, the abundance differences of 13 taxa, such as *Brevundimonas* and *Devosia*, reached statistical significance between LUSC and LUAD. The abundance differences were also observed in 63 taxa between LUSC and BPD (e.g., *Limnobacter*) and 4 taxa between LUAD and BPD (e.g., *Methyloversatilis*) (**Table S5**). Similar to the results at the phylum level, bacterial taxonomic abundances failed to show significant differences across different pathological stages of lung cancer at the genus level (**Table S5**). The taxonomic abundance comparisons at other taxonomic levels are also listed in **Table S5**. Linear discriminant analysis effect size (LEfSe) analysis was adopted to evaluate the most differently abundant taxa of each group. Linear discriminant analysis (LDA) scores over 4.0 were obtained as potential biomarkers in different pathological groups whereas LDA scores over 2.0 were utilized in lung cancer of different stages. The representative genus revealed relatively consistent results with those of abundance analysis (**Figure 4**). The LDA graph and the cladogram showed that Gammaproteobacteria was more abundant in LUSC, 14 taxa (*Brevundimonas*, Caulobacterales, Caulobacteraceae, *Brevundimonas vesicularis*, *Limnobacter*, Burkholderiaceae, unidentified Gammaproteobacteria, Alphaproteobacteria, Pseudomonadales, *Pseudomonas*, Pseudomonadaceae, *Pseudomonas xanthomarina*, unidentified Rhizobiales, and *Devosia*) were more abundant in the LUAD group, whereas 18 taxa (Sphingomonadaceae, Sphingomonadales, *Sphingomonas*, Pasteurellaceae, Pasteurellales, *Haemophilus influenzae*, *Haemophilus*, Actinobacteria, unidentified Actinobacteria, *Bifidobacterium*, Bifidobacteriales, Bifidobacteriaceae, *Bifidobacterium adolescentis*, Lactobacillales, *Lactobacillus*, Lactobacillaceae, *Blautia*, and Ruminococcaceae) were more abundant in the BPD cluster (**Figure 4A**). In the LDA graph and cladogram of lung cancer of different stages, no taxa with an LDA score over 2.0 were found in the stage III group, whereas Beggiatoales, Beggiatoaceae, Beggiatoa sp. LPN, and *Beggiatoa* were more abundant in the stage II group, Micrococcales, *Ramlibacter*, Sphingomonadaceae, and Sphingomonadales were more abundant in the stage I group (**Figure 4B**).

### 3.3 Effects of antibiotic exposure on respiratory microbiota in tissues of lung cancer and benign pulmonary diseases

To evaluate the effects of recent antibiotic exposure on respiratory microbiota in lung cancer patients, microbial taxa and diversity between lung cancer patients with different antibiotic consumption history were compared. No significant differences were observed in tissue taxonomic abundances and diversity at all taxonomic levels between lung cancer patients with and without antibiotic exposure 3 months prior to surgery (**Table S6**). For lung squamous cell carcinoma, except for one low abundant class (Chloroflexia), other bacterial taxa as well as alpha- and beta-diversity showed similar distribution between those with and without antibiotic exposure 3 months prior to surgery (**Table S6**). In terms of lung adenocarcinoma, beta-diversity was different between patients with different recent antibiotic exposure (**Table S6**). Antibiotic consumption also did not have an effect on taxonomic abundance or alpha-diversity of respiratory microbiota in lung adenocarcinoma (**Table S6**).

After excluding those who had recent antibiotic exposure, diversity and taxonomic abundance analyses were repeated to explore the role of antibiotics in shaping respiratory microbiota in lung cancer patients. Similarly, the observed OTUs and Chao1 presented a significant reduction in lung cancer groups, and no statistical significance in Shannon’s diversity index was observed. The beta-diversity analysis indicated that different clusters of microbial community existed in different lung cancer pathology (**Table S7**). Respiratory bacterial taxa at all levels failed to show significant different abundance between LUSC and LUAD. At the phylum level, the abundance of 45 taxa (e.g., Dadabacteria) was different between LUSC and BPD. The abundance of the phylum, Planctomycetes, was different between LUAD and BPD (**Table S7**). At the genus level, the abundance differences of 605 taxa, such as *Streptococcus* and *Sphingomonas*, reached statistical significance between LUSC and BPD. The abundance differences were not observed in all taxa between LUAD and BPD at the genus level (**Table S7**). Alpha-diversity and bacterial taxonomic abundances failed to show significant differences across different pathological stages of lung cancer from the phylum to the species level. With respect to beta-diversity, lung cancer stage I and III showed significant differences (**Table S7**).

### 3.4 Effects of smoking on respiratory microbiota in tissues of lung cancer

Effects of smoking history on respiratory microbiota in lung cancer patients were also evaluated. Since there was only one non-smoking LUSC case, the difference in microbiota was not compared in the LUSC group. In terms of LUAD patients, bacterial abundance and diversity of respiratory microbiota at all taxonomic levels did not show significant differences between smokers and non-smokers (**Table S8**).

## 4 Discussion

Commensal microbiota, especially those in the gut, have become potential therapeutic targets for various infectious and inflammatory diseases, as well as tumors (4, 7). Although the biomass in the respiratory tract is relatively low, the airway microbiota has been implicated in the evolution of lung cancer (13, 22, 23). However, studies focusing on relationships between respiratory microbiome and pathological types, and TNM staging, in real-world lung cancer patients are scarce. Moreover, among the respiratory microbiome, the specific taxa that participate in the pathogenesis of lung cancer remain unknown. The current study investigated the composition and diversity of respiratory microbiota in patients with benign pulmonary diseases and lung cancer. To our knowledge, this report provides new insight into the lung cancer tissue microbiota in clinical settings.

At the phylum level, similar to the study conducted by Greathouse et al., increased Proteobacteria and decreased Firmicutes and Fusobacterium were observed in lung cancer tissues compared with non-cancer population control lung tissues (24). In contrast, Actinobacteria were less frequent in the lung cancer group (especially the LUSC group). Considering the recent discovery that certain strains of Actinobacteria might produce anticancer metabolites, it is justified to further explore the association between lung cancer pathogenesis and local Actinobacteria activities in the airway (25). At the genus level, *Brevundimonas* and *Limnobacter* were abundant in all participants. Brevundimonas strains are Gram-negative, motile bacteria of the family Caulobacteraceae. In humans, Brevundimonas is an opportunistic pathogen able to cause a range of hospital-acquired infections such as bacteremia, pneumonia/pleuritis, urinary tract infection, and skin and soft tissue infection (26). Whether they are associated with cancer remains to be explored. Limnobacter was reported to be one of the potential biomarkers for hepatocellular carcinoma (27). More research on their biological role in the lung and cancer is still needed. Three genera, namely, *Streptococcus*, *Prevotella*, and *Aggregatibacter*, seemed to be enriched in LUSC cases. These genera also played a role in colon, oral cavity, and throat cancer (28–30). In particular, *Streptococcus* and *Prevotella* were also found abundant in lung cancer patients in previous studies, and certain *Streptococcus*-derived proteins could be carcinogenic by inducing epithelial inflammation (10, 28). Moreover, enrichment of *Streptococcus* and decreased *Staphylococcus* was found in the respiratory microbiome of lung cancer patients, while studies have shown that *Staphylococcus* can destroy DNA and *Streptococcus* plays a role in preventing DNA damage (31, 32). These contradictory findings may be due to the fact that the true carcinogenic taxa have not been discovered, or the same species may play different roles under different pulmonary local environmental conditions. Jungnickel et al. reported that nontypeable *H. influenzae*-associated inflammation could promote proliferation of KRAS-mutated adenomatous lesions in a TLR-dependent manner (33). However, *Haemophilus* was less abundant in the LUAD group in the present study, since nontypeable *H. influenzae* tend to colonize in the airways of patients with COPD. The low incidence of COPD in our LUAD cohort (8.89%) may explain the difference. In future studies, sequencing techniques with higher resolution (e.g., full-length 16S rRNA or metagenome) and *Haemophilus* culturomics may help elucidate the paradox. *Leucothrix* and *Sulfurovum* were mainly found in patients with lung cancer, and their activities in cancer remain unknown. *Sphingomonas* and *Bifidobacterium* were more commonly seen in patients with BPD. Increased distributions of *Sphingomonas* in non-cancerous tissues were similarly indicated in several studies, and coculture of certain strains of *Sphingomonas* and the fungus *Aspergillus fumigatus KMC-901* could produce cancer invasion inhibitory compounds (34–36). More studies are needed to clarify whether *Sphingomonas* spp. can be cancer-inhibitory. Preclinical models have shown that respiratory microbiota pattern alteration seemed to elicit inflammatory processes by upregulating several cytokines and dysregulating oxidative damage-related biomarkers (37). Oxidative stress is closely related to carcinogenesis. With respect to microbiota modulation, possible ways to combat oxidative stress include local probiotic taxon transplantation, targeting microbiota-derived pro-inflammatory metabolites and so forth. In the future, prospective interventional studies will help to elucidate the specific bacterial taxa, metabolites, and their roles in lung cancer. LEfSe analysis is commonly used to evaluate the association between microbiome and disease status (19, 38). LefSe analysis also showed that *Bifidobacterium*, *Lactobacillus*, and Ruminococcaceae, which produce short-chain fatty acids (SCFAs), are enriched in the BPD group of our study. SCFAs have been involved in inflammation alleviation, immune modulation, and anticancer effects (39, 40). Therefore, a reduction of the bacteria that generate SCFAs in the lung cancer tissue may be linked to cancer risk. Moreover, certain bacterial strains like *Bifidobacterium* spp. have been widely identified inside tumors and are capable of killing tumor cells in a natural or genetically modified form (41). The microbiome has long been reported as a reliable biomarker for the efficacy of immune checkpoint blockade therapy towards lung cancer, and antibiotic-induced microbiota dysbiosis is associated with poorer outcome following immune checkpoint inhibitor treatment (42). Mechanistically, certain bacterial epitopes might enhance the therapeutic effect of immune checkpoint inhibitors by mimicking tumor neoantigens (43). Therefore, knowing the microbiota differences between tumor and benign diseases may help recognize the potentially beneficial probiotics and epitopes, and develop new direct anti-cancer therapies as well as improve the treatment response to immunotherapy among cancer patients.

Common materials used in studies on respiratory microbiota include sputum, bronchoscopy samples, bronchoalveolar lavage fluid (BALF), and lung tissue biopsy. Due to the low-biomass nature of the lung microbiome, the respiratory samples were vulnerable to contamination. The former three types of samples are all at risk of the potential contaminants from the oral cavity. Furthermore, the specimens collected from bronchoscopy or oral sites might ignore the spatial variation of lung microbiota due to the different distribution of microorganisms in the upper and lower respiratory tract (8). Compared with BALF, lung tissue was considered as the preferred specimen to mitigate those confounding interferences and reliably reflect the local microbial composition (44). Several studies investigated the tissue microbiome of lung cancer patients, but most results came from comparisons between tumor samples and tumor-adjacent samples (22, 45–47). Since the airway space is highly dynamic, the contents in the respiratory tract, such as secretions, particles, and cells (including microbiome), are likely to spread through the lumen with the respiratory movement. As a result, the microbial compositions of the tumor-adjacent tissues may also be affected by the tumor microbiota. In this study, the control group tried to reduce those potential contaminations by using surgically removed tissue from BPD patients.

Consistent with the results of previous studies, significant reduction of alpha-diversity in respiratory microbiota of NSCLC patients was observed (13, 19, 45). Our results also revealed that bacterial beta-diversity was different between NSCLC and BPD, and beta-diversity in LUAD samples was also different from that in LUSC. These diversity differences implied that the local environment and bacterial community were different between different lung cancer pathological subtypes. The composition of the respiratory microbiome is mainly regulated by the balance of bacterial immigration, excretion, and replication (48). Reduced diversity and subsequent enrichment of certain microbes in the respiratory tract may lead to carcinogenesis and cancer evolution. For comparisons of respiratory microbial beta-diversity between NSCLC of different TNM stages, differences between stage I and stage III were significant, suggesting that the local airway environments changed with different cancer development stages. Kim et al. indicated that declined alpha-diversity was found in lung cancer of more advanced stages (III–IV). However, no such difference was found in our study (49). Moreover, similar to results of most microbiota studies, whether these microbiota changes are the cause, mediator, or result of cancer evolution and what the specific cancer-promoting component(s) in respiratory microbiota are remain unknown. Due to the limited sample size, more transregional multi-center studies are necessary to further validate those differences.

Antibiotics are considered as broad-spectrum manipulators of commensal microbiota. In the current comparisons of lung cancer, bacterial diversity among recent usage of antibiotics, except for the beta-diversity of respiratory microbiota in lung adenocarcinoma, showed significant difference, and both the alpha-diversity and taxonomic abundances showed similar results across different pathologies. After excluding lung cancer patients with recent antibiotic exposure, more taxa with significantly different abundances were revealed between lung cancer patients and patients with benign pulmonary diseases. Observational studies indicated that antibiotic use was an independent risk factor for lung cancer occurrence. Antibiotic exposure might promote carcinogenesis *via* disruption of commensal microbiota, which could contribute to dysregulation of host immune homeostasis (50). Our results supported the idea that short-term antibiotic consumption seemed to be a subsidiary rather than a dominant power in molding the composition of airway microbiota. Antibiotics might affect the community structure of respiratory microbiota to some extent. However, because different types and doses of antibiotics may have varied effects on bacteria, more detailed work is necessary to clarify how antibiotics shape the commensal microbiota and whether these changes have a role in lung cancer evolution. Furthermore, current methods for precisely modulating the gut microbiome mainly include fecal microbiota transplantation, probiotics and prebiotics, and diet control. Similar new techniques, such as respiratory microbiota transplantation and bacteriophages, are necessary to elucidate the role of certain individual bacterial taxon in lung cancer evolution (51).

Cigarettes may modify commensal microbiome by microbes in tobacco, tobacco-induced defect of antimicrobial defenses, increased biofilm formation by certain advantageous bacteria, or altered local microenvironment (e.g., oxygen tension) (52). Yu et al. indicated that chronic exposure to tobacco smoke was associated with higher diversity of lung microbiota and the higher proportion of smokers in LUSC may be the explanation (53). Long-term inhalation of tobacco-related detrimental particles could increase the airborne spread of microbes and impede the elimination of microbes from the respiratory system (53). As a result, increased microbial burden might be found in smokers. However, Greathouse et al. and our group found no significant changes in alpha-diversity by smoking status. For LUSC patients, Greathouse et al. showed that the relative abundance of *Acidovorax* was higher in former and current smokers (24). Zheng et al. reported that increased *Pseudoalteromonas* sp. *CF149*, *Roseburia hominis*, *Penicillium expansum*, etc. and decreased *Pseudomonas mosselii* and *Pseudomonas putida* were shown in mixed samples of bronchoscopy and lobectomy in NSCLC patients with a smoking history (19). The following reasons may help elucidate those differences of taxonomic relative abundance with different tobacco exposure. Firstly, previous studies included both LUSC and LUAD in the analyses, and the greater proportion of smokers in the LUSC group may explain the differences. LUAD mainly arises from peripheral airways, and its association with smoking is weaker compared with LUSC. As a result, the effect of cigarettes on the respiratory microbiome of LUAD may be alleviated. Secondly, increased microbial burden in smokers does not necessarily mean higher diversity. Being continuously exposed to the external environment, the respiratory microbiome undergoes dynamic changes. The immigrant microorganisms could become part of the resident microbiota, but the majority belongs to the transient group, which could not grow in the respiratory tract and contribute little to respiratory microbial diversity (48). Moreover, different sample types and processing procedures might also contribute to the variations. The exact causal relationship between cigarettes and microbiome alteration in lung cancer patients remains to be further explored (52).

There are several potential limitations in the current study. First, it is difficult to balance baseline characteristics among different groups due to the disease nature and consecutively enrolled cohort. As a result, the inter-group age, gender, smoking status, degree of differentiation, and TNM stage did not match. Alterations of airway microbiome with respect to those characteristics were not evaluated. Second, the limited sample size, especially the BPD group, might compromise the power of this study. In the comparison of taxonomic abundance among different pathological stages of lung cancer, no bacterial taxa showed significant difference. Whether microbial composition differs among lung cancer stages remains to be explored. Studies with sufficient and evenly distributed samples are warranted to further validate the current results. Third, this study was cross-sectional and unable to determine the temporal relationship of the respiratory microbiome to onset or progression of lung cancer. Fourth, 16S rRNA amplicon sequencing is liable to contamination during PCR procedures and generally classifies dominant bacteria to the genus level. Shotgun metagenomic sequencing may help make up these defects and identify more specific species within these genera for each lung sample (54). Fifth, OTU-level flow, Mothur, was still used in our study to be comparable to studies about respiratory microbiota in lung cancer patients. ASV (amplicon sequence variant)-level pipelines (DADA2, QIIME2-Deblur, and USEARCH-UNOISE3) are justified to confirm the current results. In addition, 16S rRNA sequencing cannot provide information on the viability of bacteria and bacteria–host interactions. Bacterial culture, respiratory microbiota transplantation, and metabonomics are potential ways to clarify the role of flora activities in NSCLC.

In conclusion, we found significantly different respiratory microbiome taxa, abundance, and diversity in lung cancer of different pathology and some stages. Short-term antibiotic application might play a minor role in molding airway microbiota in lung cancer patients. Composition and diversity of respiratory microbiota in lung adenocarcinoma are not affected by cigarette exposure. Prospective longitudinal studies that utilize bacterial culture, respiratory microbiota transplantation, and metabonomics are needed to further clarify the role of respiratory microbiota activities in NSCLC.

## Data availability statement

The datasets presented in this study can be found in online repositories. The names of the repository/repositories and accession number(s) can be found below: BioProject, accession number PRJNA834019.

## Ethics statement

The studies involving human participants were reviewed and approved by the Institutional Review Board committee of West China Hospital, Sichuan University. The patients/participants provided their written informed consent to participate in this study.

## Author contributions

Study conceptualization and design: YH, XZ. Supervision and administrative support: YH. Funding acquisition: XZ. Specimen processing and data collection: XZ, XL. Data analysis and interpretation: all authors. Manuscript writing and editing: all authors. All authors contributed to the article and approved the submitted version.

## Supplementary material

SUPPLEMENTARY TABLE S1 The detailed software and the corresponding parameters used in the 16S rRNA amplicon analysis.

SUPPLEMENTARY TABLE S2 Relative abundance of the top 30 respiratory taxa at the phylum (A) and genus (B) levels by pathological statuses (LUSC vs. LUAD vs. BPD).

SUPPLEMENTARY TABLE S3 Anosim analysis between different pathology and lung cancer TNM stages.

SUPPLEMENTARY TABLE S4 Comparisons of bacterial floral alpha and beta diversity between different pathology and lung cancer stages. LCL and UCL refer to lower confidence limit and upper confidence limit in the Wilcoxon test, respectively.

SUPPLEMENTARY TABLE S5 Comparison of the respiratory bacterial taxa between different lung cancer pathology and TNM stages from phylum to species level.

SUPPLEMENTARY TABLE S6 Comparison of respiratory bacterial taxa and diversity between lung cancer with and without antibiotic exposure.

SUPPLEMENTARY TABLE S7 Comparison of respiratory bacterial diversity and taxa between different lung cancer pathology and stages without antibiotic exposure.

SUPPLEMENTARY TABLE S8 Comparison of respiratory bacterial taxa and diversity between lung adenocarcinoma with and without smoking history. Only one case in LUSC group had no smoking history, therefore bacterial taxa and diversity of smokers and non-smokers in LUSC group were not compared.
